# The Upper Triassic deposits of the west Bangong-Nujiang suture zone and their paleogeographic implications

**DOI:** 10.1038/s41598-021-98257-5

**Published:** 2021-09-30

**Authors:** Guichun Wu, Zhansheng Ji, Gary G. Lash, Jianxin Yao

**Affiliations:** 1 Institute of Geology, Chinese Academy of Geological Sciences, Beijing, 100037 China; 2 Chinese Academy of Geological Sciences, Beijing, 100037 China; 3 Department of Geology and Environmental Sciences, State University of New York - Fredonia, Fredonia, NY 14063 USA

**Keywords:** Solid Earth sciences, Geology, Stratigraphy

## Abstract

The Bangong-Nujiang Suture Zone (BNSZ) of Tibet (Xizang) has been interpreted to represent a relic of the Bangong-Nujiang Ocean. However, the existence of this ocean during Triassic time remains a point of contention. A sedimentary succession spanning the Upper Permian through Triassic described from the central BNSZ suggests that the Lhasa and South Qiangtang terranes were contiguous thus negating the existence of a terrane-separating ocean during Triassic time. However, the apparent lack of Triassic deposits in the west BNSZ has called into question the existence of Triassic deposits in the central region of the BNSZ. Our biostratigraphic work in the Wuga Formation of the Gaize area has yielded abundant Norian conodonts thus confirming the existence of Upper Triassic deposits in the west BNSZ. The clastic deposits of the Wuga Formation are herein interpreted to be of Rhaetian age. Moreover, intercalated limestone and chert are termed the Dongnale Formation of Norian age. The Norian to Rhaetian succession can be correlated with strata of the central BNSZ as well as with deposits of the Lhasa Terrane and the South Qiangtang Terrane. Similar stratigraphies among these regions through the Late Triassic suggests a shared depositional setting and that the BNSZ was not an ocean in Norian and Rhaetian time.

## Introduction

**Figure 1:**
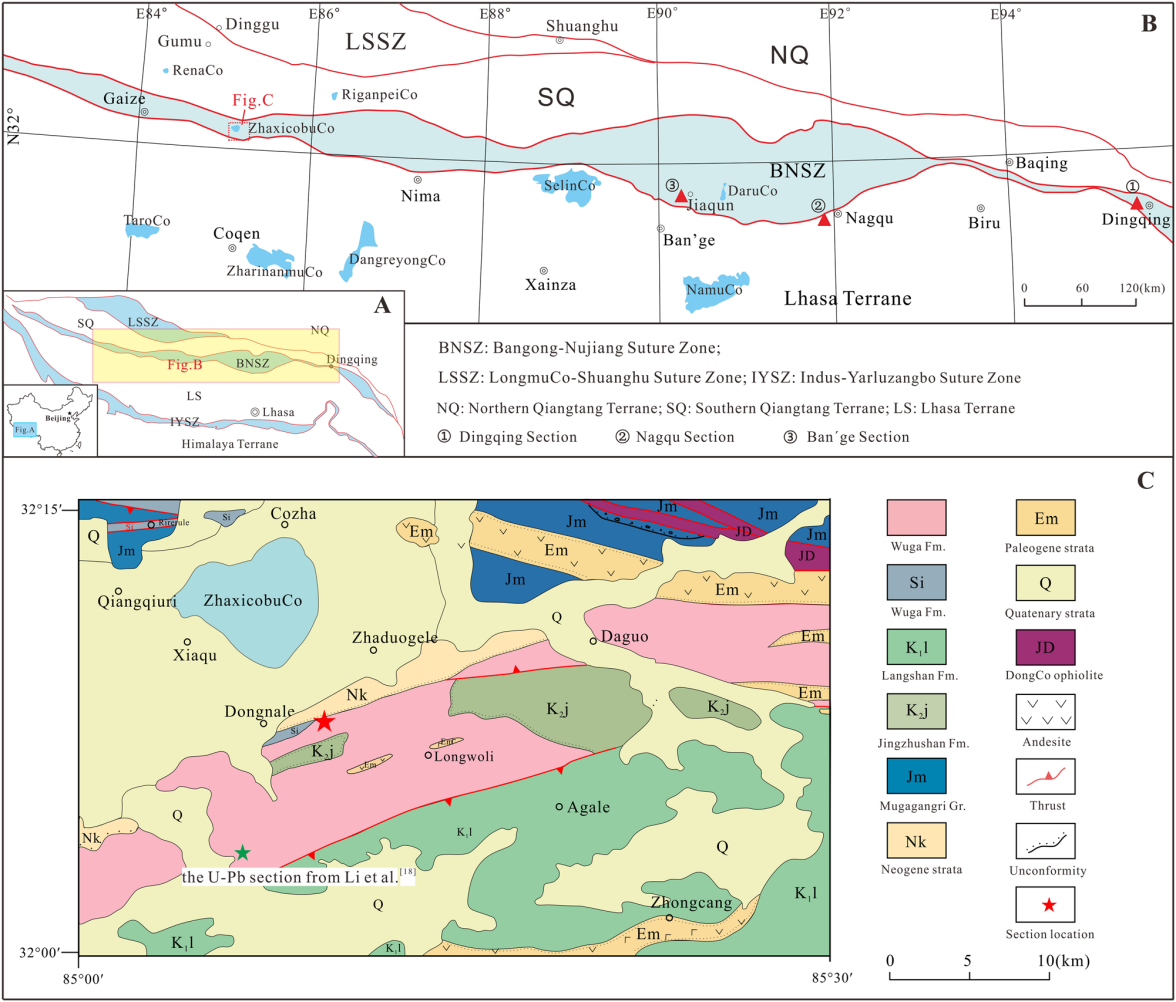
(**A**,**B**) (based on Pan et al.^3^) shows the main tectonic terranes of Tibet; (**C**) (modified after Zeng et al.^17^) geologic map displaying details of the study area.

The Bangong-Nujiang Suture Zone (BNSZ) is an important tectonic element of Tibet^1–4^ (Fig. 1). Some palaeogeographic reconstructions suggest that the 2400 km-long BNSZ was a geographic barrier, the Bangong-Nujiang Ocean, that separated the Lhasa Terrane in the south from the South Qiangtang Terrane to the north^5–7^. However, there is little agreement regarding the age of the inferred ocean and even its existence during Triassic time^2,8–10^. The debate centers on three opinions regarding the tectonic significance of the BNSZ. One view holds that the BNSZ was not represented by an ocean during the Triassic^6,8,11,12^. A contrary argument maintains that the BNSZ was a long-standing ocean that existed from Late Paleozoic to Cretaceous time^2,5^. A third opinion holds that the ocean existed briefly in Late Paleozoic time, closed and then re-opened during Middle to Late Triassic time^13,14^. It is clear, then, that elucidating the Triassic sedimentary history of the BNSZ is critical to unraveling the paleogeographic evolution of this region of Tibet.

Lower and Middle Triassic deposits have generally been considered to be poorly represented or even absent in the BNSZ^15^. Indeed, only the clastic deposits of the Quehala Formation of the central region of the BNSZ and the Wuga Formation in the west are considered to have accumulated in Late Triassic time^15^. However, more recent biostratigraphic results stemming from an analysis of radiolarians, conodonts, and corals suggests that Triassic deposits are more widespread within the BNSZ, especially its central and eastern regions, than heretofore believed (Fig. 1B). These deposits include cherty strata that host Carnian radiolarians and conodonts described from the Dingqing area^16^; chert-dominated deposits containing Ladinian radiolarians in the Gajia area^14^; and dolomite-dominated rocks bearing Late Permian corals and Early Triassic to Anisian conodonts and limestone that yields Norian corals documented from the Ban’ge area^12^. These findings confirm that Triassic deposits are well represented in the central and east BNSZ (Fig. 1). The marked similarity of the Triassic succession of the central BNSZ with deposits of the Lhasa and Qiangtang terranes suggests that the BNSZ was an extension of these terranes rather than a geographic barrier separating them in Late Permian to Triassic time^12^.

The above-described biostratigraphic data have been recovered primarily from the central and east part of the BNSZ. The biostratigraphy of the west region of the BNSZ remains unresolved. Although the Wuga Formation has been regarded by some to be Late Triassic in age and equivalent to the Quehala Formation of the central BNSZ^17^, U–Pb age data from sandstone of the Wuga Formation reveals it to be of Late Jurassic to Early Cretaceous age^18^ (Fig. 1C). In order to demonstrate the existence of Triassic deposits in the west BNSZ, we carried out a biostratigraphic investigation of the Wuga Formation east of Gaize (Fig. 1B). Our goal is to elucidate the Triassic nature of the BNSZ in its western region.

## Geological setting

The study area is located near ZhaxicobuCo Lake, east of Gaize County (Fig. 1B). Tectono-stratigraphically it belongs to west part of the BNSZ (Fig. 1). The Late Triassic deposits of the BNSZ in the study area have been assigned to the Wuga Formation^17,18^. These deposits are disconformably overlain by conglomerate of the Cretaceous Jingzhushan Formation (K_2_j) and locally the Neogene Kangtuo Formation (Nk) (Fig. 1C). Neogene volcanic rocks also crop out in the region (Fig. 1C). To the south, the Wuga Formation is in fault contact with the Lower Cretaceous Langshan Formation (K_1_l), a limestone-dominated succession. It is noteworthy that the fault separating the Langshan and the Wuga formations is considered to be the southern boundary of the BNSZ in the study area (Fig. 1C). The DongCo ophiolites crop out to the north of the study area. No direct contact relationships between the Wuga Formation and the ophiolites were observed.

The 1:250,000 geological map survey^17^ describes the Wuga Formation as being composed of clastic rocks intercalated with limestone and chert. The presence of bivalve fossils interpreted to be *Protocardia* sp. collected from similar deposits not far from the study area suggests a Late Triassic age^17^. Moreover, a succession of deep-water shale and sandstone near Dongnale village has also been assigned to the Wuga Formation though fossil evidence has not been recovered (Fig. 1C). In summary, the Wuga Formation is a stratigraphically complicated unit that has yielded rare fossils precluding regional correlation with other sedimentary units. Our biostratigraphic investigation of the Wuga Formation was carried out near the Dongnale Village (Fig. 1C) with the goal of providing the necessary fossil evidence for or against the existence of Triassic deposits in the western region of the BNSZ.

## Results

**Figure 2:**
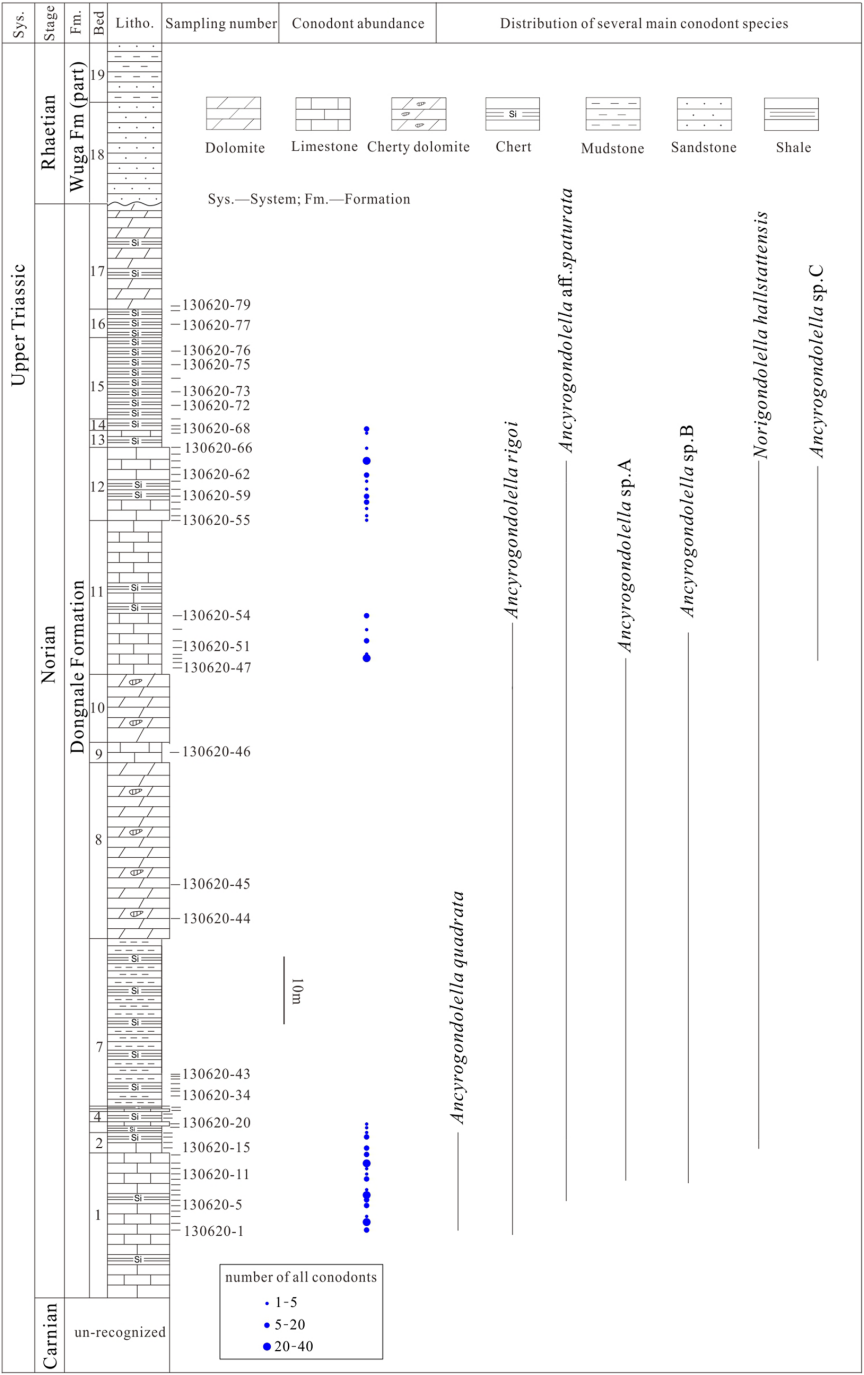
Lithologic log of the ZhaxicobuCo section illustrating the sample locations of conodonts and the principal conodont species.

**Figure 3:**
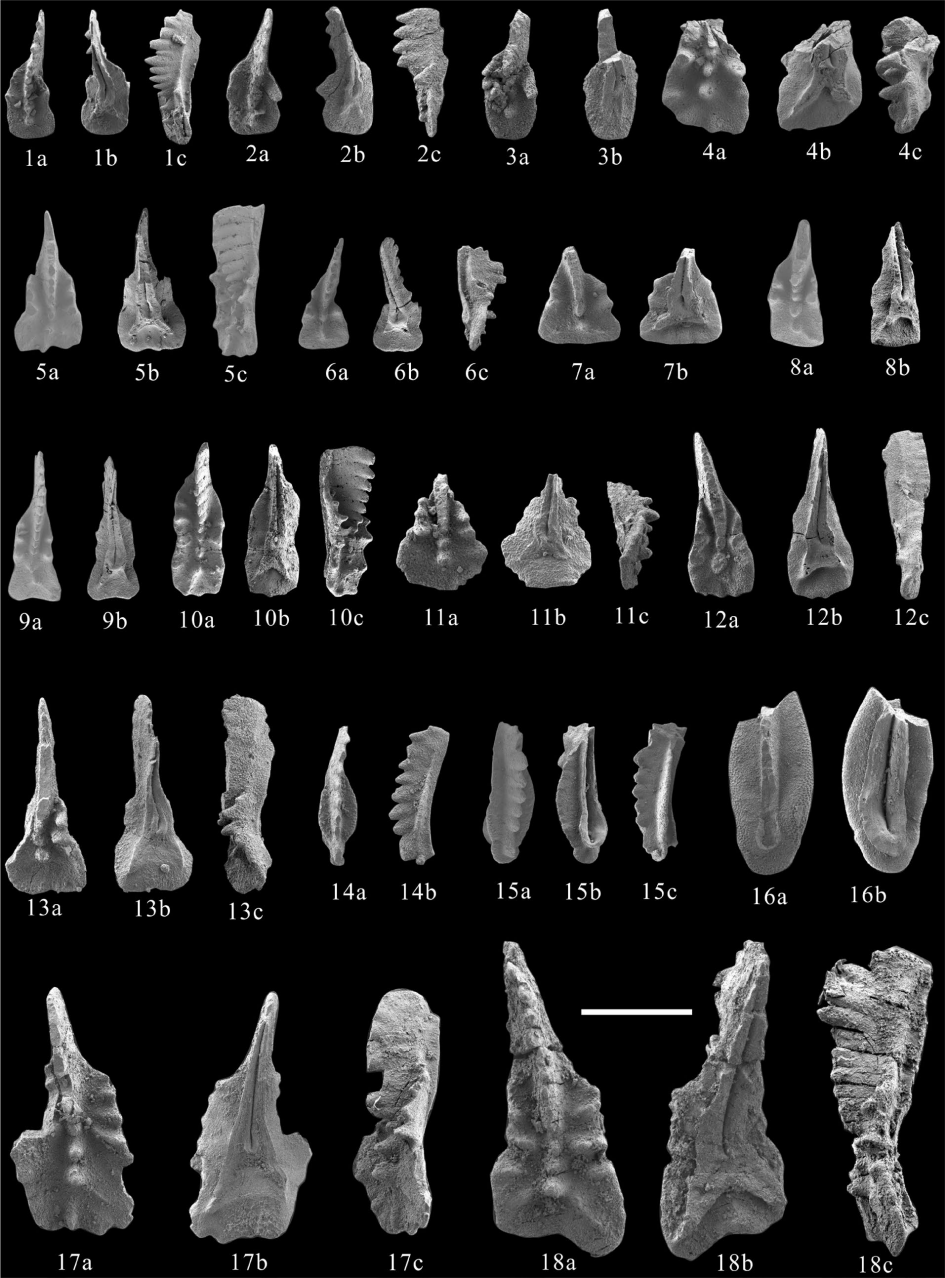
Norian conodonts recovered from the ZhaxicobuCo Section. Scale bar is 400 μm. **1–3**, *Ancyrogondolella* sp. **A.** 1, 130,620–10; 2, 130,620–12; 3, 130,620–49; **4–7**, **18,** *Ancyrogondolella rigoi*. 4, 130,620–52; 5, 130,620–17; 6, 130,620–64; 7, 130,620–63; 18, 130,620–52; **8–9**, *Ancyrogondolella quadrata*, 8, 130,620–1; 9, 130,620–10; **10**, *Ancyrogondolella* sp. **B,** 130,620–17; **11**, *Ancyrogondolella* sp. **C,** 130,620–49; **12–13, 17**, *Ancyrogondolella* cf. *rigoi*. 12, 130,620–10; 13, 130,620–49; 17, 130,620–12; **14–16**, *Norigondolella hallstattensis*, 14, 130,620–64; 15–16, 130,620–62.

The studied ZhaxicobuCo stratigraphic section comprises 19 beds (Fig. 2). The interval of the section spanning Beds 1 to 17 is composed of limestone, cherty dolomite, and chert. Overlying strata is dominated by sandstone and mudstone (Fig. 2). The contact of Beds 17 and 18, the inferred contact of the Dongnale Formation and overlying Wuga Formation, is interpreted to be a disconformity (Fig. 2). Of 79 collected samples, 34 yielded conodonts (Fig. 2). Where collected, conodonts are abundant in number (Fig. 2) and diverse though only the most common species are reported here. Detailed faunal data will be published elsewhere. Age-indicative species include *Ancyrogondolella rigoi and Ancyrogondolella quadrata* (Figs. 2 and 3). It is noteworthy that these species have been documented from Lower Norian stage strata of Greece^19^, Hungary^20^, Italy^21–23^, Slovenia^24^, Japan^25,26^, and Canada^27–29^. Therefore, strata of Beds 1 to 17 of the Dongnale section confirm the presence of Norian age deposits in the west BNSZ.

## Discussion

**Figure 4:**
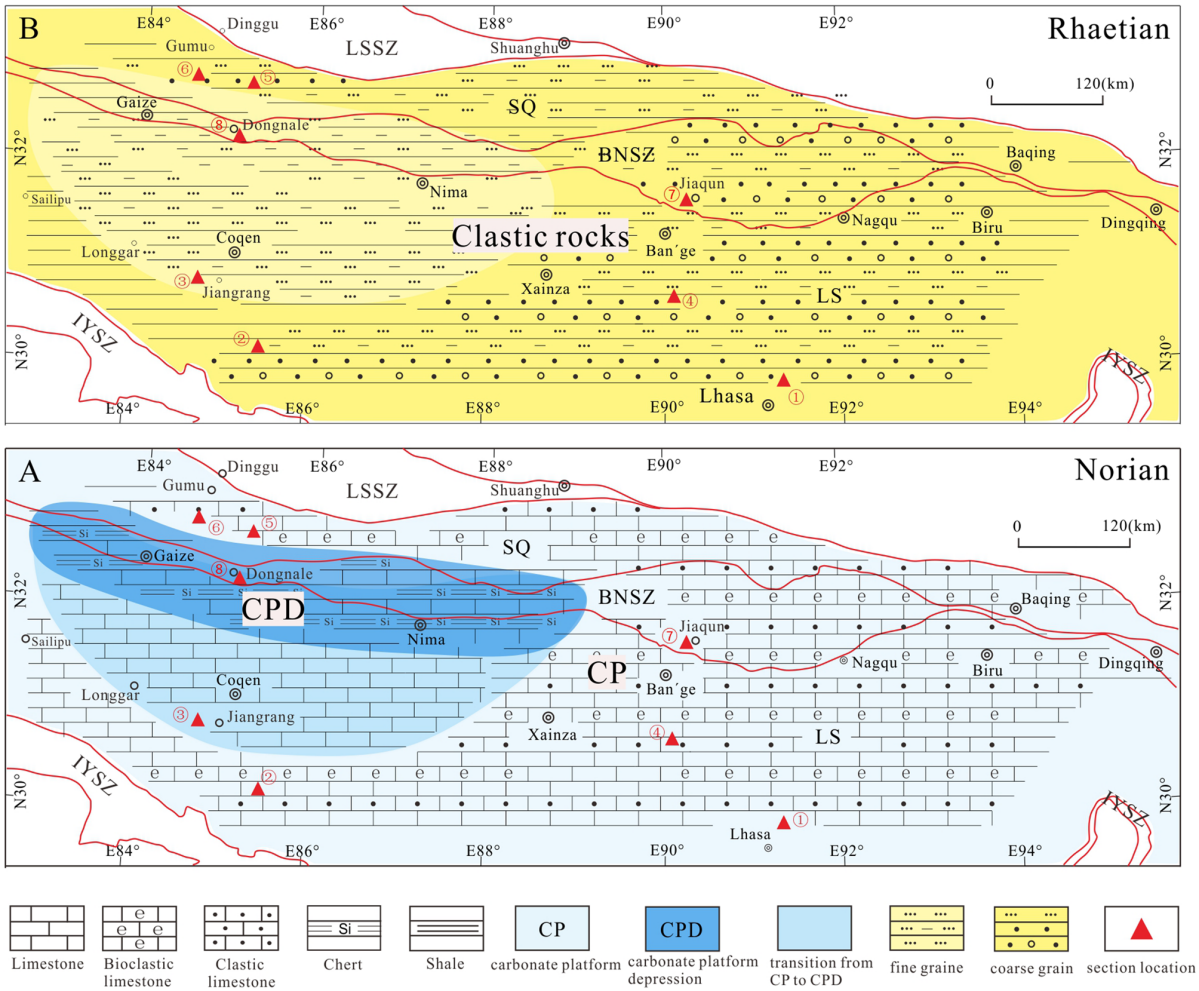
The Norian (A) and Rhaetian (B) sediments in the BNSZ, Lhasa Terrane, and South Qiangtang Terrane. ①-Mailonggang section (conodonts) ^30^; ②-Yawa section (conodonts and corals) ^32^; ③-DibuCo section (conodonts) ^31^; ④-NamuCo section (corals)^33^; ⑤,⑥-sections within the South Qiangtang Terrane (corals)^34^; ⑦-DaruCo section (corals)^12^; ⑧-ZhaxicobuCo section (conodonts; this paper); *LS* Lhasa Terrane, *SQ* South Qiangtang Terrane, *LSSZ* LongmuCo-Shuanghu Suture Zone, *BNSZ* Bangong-Nujiang Suture Zone, *IYSZ* Indus-Yarlung Zangbo Suture Zone. Red solid lines define boundaries between different tectonic terranes.

As noted above, the existence of Upper Triassic deposits in the west BNSZ has been debated in previous research^17,18^. However, our collection of Norian conodonts from the ZhaxicobuCo section establishes the presence of Upper Triassic deposits in the west BNSZ. Intercalated limestone and chert of the newly recognized Norian succession together with the overlying clastic deposits have been assigned to the Wuga Formation^17,18^. Herein, however, we separate the Norian deposits from the originally described Wuga Formation based on marked lithologic differences and because the former are disconformably overlain by the sandstone and mudstone succession, what we now refer to as the Wuga Formation (Fig. 2). Regionally, the revised Wuga Formation correlates with the Rhaetian Quehala Formation of the central BNSZ, a clastic succession. Norian rocks have been described from the central BNSZ (Mailonggang Formation)^12^, the Lhasa Terrane (Mailonggang Formation and Jiangrang Formation)^30–33^, and the South Qiangtang Terrane (Riganpeico Formation)^34^ where they are made up largely of thick-bedded limestone and dolomite (Fig. 4). However, the newly recognized Norian deposits are composed of thin-bedded limestone and chert, implying deposition in a deeper water setting than Norian rocks from adjacent regions. Thus, we propose that the Norian deposits of the west BNSZ be referred to as the Dongnale Formation for the village proximal to the studied section (Fig. 2).

The Norian and Rhaetian sequence described in the present study correlates with that of the central BNSZ, Lhasa Terrane and the South Qiangtang Terrane (Fig. 4). Still, there are meaningful differences among the successions. For example, strata of the west BNSZ appear to have accumulated in a deeper marine setting than equivalent deposits of other regions. Indeed, the Dongnale Formation contains abundant cherty rocks whereas the Mailonggang/Jiangrang/Riganpeico sequence from other regions is dominated by limestone and lacks chert. Similarly, Rhaetian sandstone of the Wuga Formation is finer grained than are Rhaetian deposits of other regions (Fig. 4). Nevertheless, the sharp change up-section from Norian fine-grained carbonate marine deposits to Rhaetian clastic rocks as well as the separating disconformity may reflect the effects of the Indosinian Orogeny in Tibet. It is noteworthy that the Triassic Indosinian Orogeny had been differentiated into Early to Middle Triassic and Late Triassic phases^35^. The latter is considered to represent the main orogenic episode^36^. Evidence of the Early to Middle Triassic phase of the Indosinian Orogeny in Tibet is not obvious as Lower and Middle Triassic rocks are dominated by marine deposits. Indeed, the disconformity at the base of the Upper Triassic clastic succession is considered to be a signature of the Late Triassic orogenic phase in the Lhasa Terrane^37^ and east of Qiangtang Terrane^35^. Similarly, the disconformable contact of Norian and Rhaetian deposits of the newly described ZhaxicobuCo stratigraphic section likely reflects the effects of the Indosinian Orogeny in the study region.

## Palaeogeographic implications

**Figure 5:**
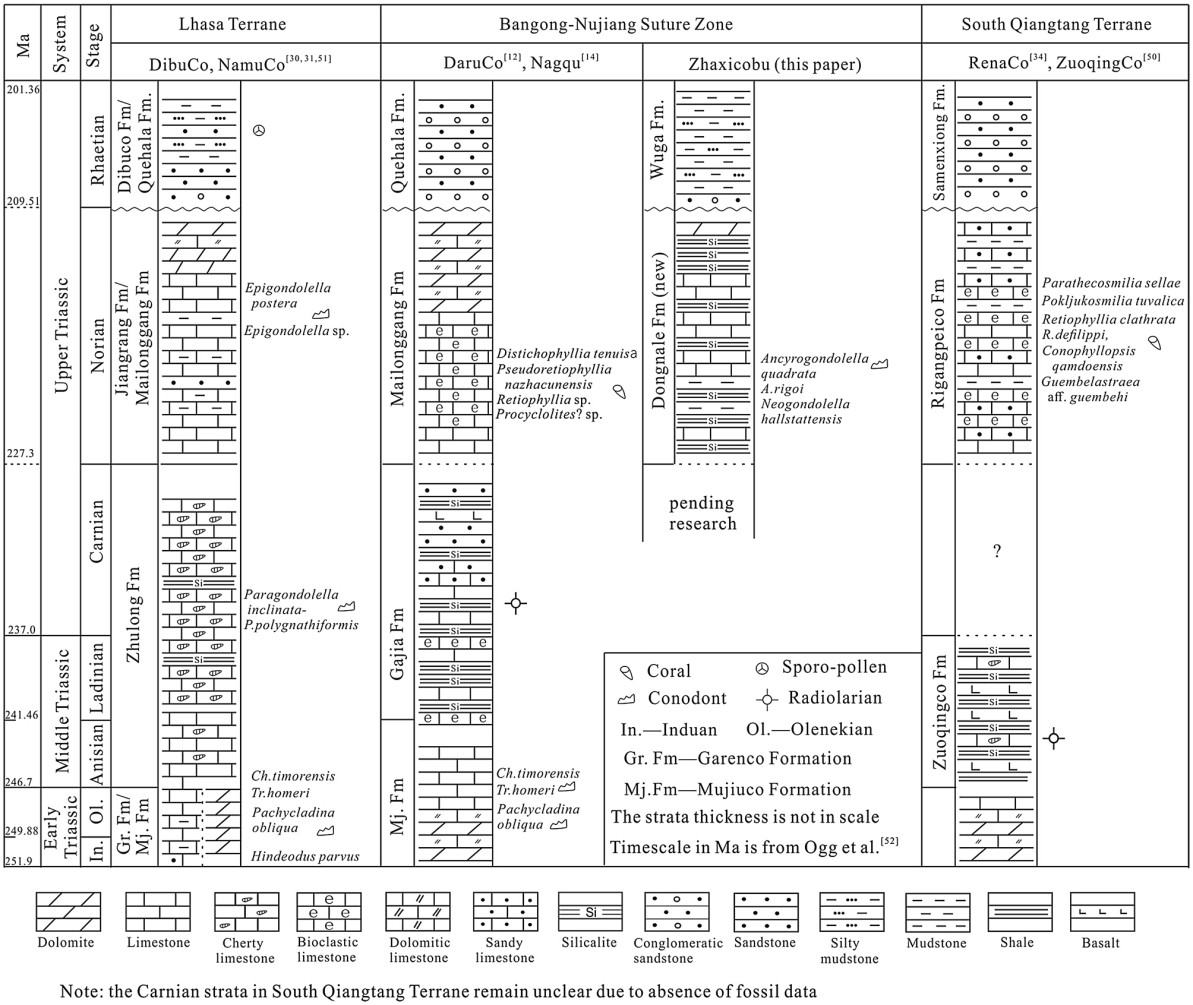
Triassic sedimentary successions described from the BNSZ, Lhasa Terrane, and South Qiangtang Terrane. The main fossil taxa are marked beside each column.

As noted earlier, the timing of the opening of the inferred Bangong-Nujiang Ocean and its existence during Triassic time remain controversial. Most age data from ophiolites distributed throughout the BNSZ show that the ocean was of short duration and started to open perhaps as early as Early Jurassic time^8,10,38–40^. A Re-Os age of 254 ± 28 Ma from harzburgites in the west BNSZ fixes the opening time as Late Paleozoic^41^ though the tectonic significance of this data remains uncertain^39^. Thus, there is no reliable ophiolite data at present that would suggest that the Bangong-Nujiang Ocean existed during the Triassic. The dearth of palaeomagnetic research from the South Qiangtang Terrane make it difficult at best to decipher the relationship of the Lhasa Terrane and the South Qiangtang Terrane^42^, and data from the Lhasa Terrane are mainly from Early Triassic deposits^43,44^. The paleogeographic interpretation of these terranes depends largely on the palaeo-position of the North Qiangtang Terrane^42^. Palaeomagnetic data from the North Qiangtang Terrane are much complete, including that from Early Triassic and Late Triassic rocks though interpretations are open to debate. For example, the cited Early Triassic paleo-latitude value is 10.6° but the position relative to the paleo-equator (i.e., north hemisphere versus south hemisphere) remains a disputed topic ^42,43,45^; it appears to have occupied a northern hemisphere position in Late Triassic time though estimated paleo-latitude positions display wide variation, including 17.9° N^43^, 27° N^46^ and 34.1° N^47^.In addition to aiding in the establishment of relative ages and the correlation of stratigraphic units, biostratigraphic data can provide valuable information useful to palaeogeographic reconstruction, including recognition of displaced terranes^48^. The association of Jurassic ophiolites, Late Triassic bivalves and the coal bearing clastic deposits in Dingqing area have been interpreted as evidence that the Bangong-Nujiang Ocean was not open during Late Triassic time^38^. More recent biostratigraphic data^12,33,34^ appears to confirm this opinion. As noted earlier in this paper, the robust lithologic similarity of Triassic rocks of the central BNSZ with correlative rocks of the Lhasa Terrane to the south and the South Qiangtang Terrane to the north suggests that the former was not a geographic barrier separating the latter terranes during the Triassic^12^ (Fig. 5). Specifically, the Early Triassic history of the three paleogeographic regions entailed accumulation of dolomite-dominated successions that have yielded conodont specimens of *Pachycladina* that thrived in shallow water, low-latitude regions^12,49^. Moreover, limestone-dominated Norian strata of these regions host common Tethyan corals^12,34^. These observations offer further compelling evidence that the BNSZ was not a geographic barrier during Early Triassic and Norian to Rhaetian time. The conodont-bearing Norian rocks of the Gaize area confirm the existence of Upper Triassic deposits in the west BNSZ. Further, the studied ZhaxicobuCo Norian-Rhaetian succession displays a depositional history similar to that of correlative sections described from the central BNSZ, the Lhasa Terrane, and the South Qiangtang Terrane (Fig. 5). These regions were elements of an extensive Norian carbonate platform containing local depressions. Late Triassic uplift induced by the Indosinian Orogeny is marked by the widespread disconformable contact of Norian and Rhaetian strata (Figs. 2, 4, and 5). Thus, similar stratigraphies and fossils among the three palaoegeographic regions confirm that the BNSZ was not an oceanic barrier during Late Triassic time.

It is noteworthy the Middle Triassic sediments accumulated in the deeper marine setting compared to Early Triassic and Late Triassic strata (Fig. 5). Moreover, Middle Triassic cherty rocks associated with basalt of the central BNSZ^14^ and north margin of the South Qiangtang Terrane^50^ have yielded radiolarians. These observations suggest that Tibet may have experienced crustal extension during Middle Triassic time with the BNSZ and the north margin of the South Qiangtang Terrane separately being the axis of extension (Fig. 5). The timing of the inferred episode of crustal extension may coincide with the ages of the ophiolites described from within and proximal to the BNSZ. Whether or not the BNSZ served as a palaeobiogeographic barrier during Middle Triassic time requires further work.

## Conclusions

1. The collection of Norian conodont species *Ancyrogongdolella rigoi* and *A. quadrata* from deposits of the Gaize area confirms the presence of Upper Triassic strata in the west BNSZ. The conodonts were collected from a marine succession dominated by intercalated limestone and chert herein named the Dongnale Formation.
2. Results of the present study establish the presence of an Upper Triassic succession in the west BNSZ that includes, in ascending order, the newly named Norian Dongnale Formation and clastic rocks of the Rhaetian Wuga Formation.
3. The newly recognized Upper Triassic succession establishes the presence of Triassic deposits along the 2400-km-long BNSZ. Lithologic and faunal similarities of Upper Triassic deposits of the BNSZ, Lhasa Terrane, and the South Qiangtang Terrane suggest that the former was not an oceanic barrier that separated the latter two palaeogeographic regions during Late Triassic time.

## Systematic description

***Ancyrogondolella*** **Budurov, 1972**

**Diagnosis.** This genus has a generally broad platform with variably ornamented denticles on the platform margins. The cusp is usually the smallest one and lies close to the central platform. The keel on the lower surface bifurcates close to or posterior of the subcentral or anteriorly shifted pit.

**Remarks.** The genus may be the ancestor of *Epigondolella, Mockina*, and *Orchardella*^28^. It differs from these genera by having a bifurcated keel.

*Ancyrogondolella quadrata* Orchard, 1991

Figures 3.8–3.9

1983 *Epigondolella abneptis* subsp. A population—Orchard^53^, p. 179–181, fig. 4.

1991 *Epigondolella quadrata* n. sp.—Orchard^54^, p. 311, pl. 2, figs. 1–3, 7–9.

2012 *Epigondolella quadrata* Orchard—Mazza et al.^22^, Pl. 5, figs. 2–10.

2014 *Epigondolella quadrata* Orchard—Orchard^55^, p. 55–57; fig. 40/10–18, 19–27.

2020 *Ancyrogondolella quadrata* Orchard (Orchard, 1991)-Karadi et al.^24^, figs. 5/11.

**Description.** The P_1_ elements of this species display a sub-rectangular platform with generally parallel lateral margins and a length-to-breath ratio of between 1.5:1 and 2:1. A medial constriction may occur on both sides. Two to three denticles are present on each side of the anterior platform. The nearly centrally located small cusp is usually followed by two to three large denticles. Towards one postero-lateral corner from the last denticle, an accessory ridge-like carina may be present. The free blade is between 1/2 and 1/3 element length. The pit is almost centrally located, and the posterior keel usually bifurcates immediately posterior of the pit or a short distance beyond it.

**Remarks:** Based on the posterior platform shapes, Orchard^55^ differentiated two morphotypes for the species *E. quadrata*. The alpha morphotype has acutely angled postero-lateral corners with an expanded posterior platform margin whereas the beta morphotype has a subrectangular posterior platform with subparallel lateral margins. The two mophotypes could be recognized in the study area. Figure 3.8 is the beta morphotype and fig. 3.9 is alpha morphotype.

**Materials**. 22 specimens from 8 samples.

*Ancyrogondolella rigoi* Kozur, 2007

Figures 3.4–3.7, 3.18.

2007 *Epigondolella rigoi* Kozur n. sp.-Noyan and Kozur^19^, p.167, fig. 6.2–6.5.

2007 *Epigondolella rigoi* Kozur n. sp.-Moix et al.^56^, p.293.

2012 *Epigondolella rigoi* Noyan and Kozur -Mazza et al.^22^, p.108, pl. 6, figs. 1–7.

2018 *Epigondolella rigoi* Kozur in Noyan and Kozur-Karadi^20^, p.162, Pl. 1, fig. 5.

2020 *Epigondolella rigoi* Kozur in Noyan and Kozur-Karadi et al.^24^, figs. 5/12–13.

**Description.** The P_1_ elements of this species have a sub-triangular platform that is obviously expanded posteriorly. The platform is relatively short and displays with a length-to-breath ratio of between 1:1.2 and 1.3:1. The anterior lateral margins yield one to four denticles on each side. The posterior platform is usually flat and unornate. It is occasionally wavy but never displays denticulation. The small cusp is more or less beneath the medial platform, and is usually followed by 1–2 large nodes. Laterally the element is straight and slightly stepped. The pit lies beneath the central platform, and the posterior keel usually bifurcates immediately posterior of the pit or a short distance beyond it.

**Remarks.***Ancyrogondolella triangularis* also has sub-triangular platform shape and bifurcated keels, but differs in having a strong denticulation in the posterior margin. *Ancyrogondolella quadrata* has similar marginal denticulation but its platform is not so expanded as in *Ancyrogondolella rigoi*.

**Materials.** 30 specimens from 12 samples.

*Ancyrogondolella* cf. *rigoi* Kozur, 2007

Figures 3.12–13, 3.17.

**Remarks**. The P_1_ elements of this species are much similar to *Ancyrogondolella rigoi* in having a sub-triangular platform that is expanded posteriorly, a centrally located cusp that is followed by a large denticle, two to three large denticles in anterior half of the platform margins, and an unornate, occasionally wavy posterior platform margin. However, our specimens differ in having deeper adcarinal furrows. Besides, the keel on the lower side is not immediately bifurcated posterior of the centrally located pit but maintains expanded till close to the posterior end of the platform. Only a slight bifurcation occurs at the keel end.

**Materials.** 5 specimens from 3 samples.

*Ancyrogondolella* sp. A

Figures 3.1–3.3

**Remarks.** The P_1_ elements of the present specimens have a relatively short platform with length-to-breadth ratios of 1.3:1 to 1.6:1. Platform margins maintain constant width for half of the unit length. The posterior-lateral corners are broad round. One or two large and sharp denticles are present on both sides of the anterior platform. The posterior half of the platform margin is unornate. The medially located small cusp is followed by a large denticle that ends the carina. There is a wide distance between the last denticle and the posterior platform end. In the lower view, the pit is somewhat centrally located in the platform. The keel is bifurcated posterior of the pit. The specimens are different from *Ancyrogondolella quadrata* in that the posterior margin is round rather than square.

**Materials.** 8 specimens from 4 samples.

*Ancyrogondolella* sp. B

Figure 3.10.

**Remarks.** The P_1_ elements of the present specimens are characterized by having a very long platform, with a length-to-breath ratio up to 2:1 and parallel margins. Prominent denticles are evenly spaced on both lateral platform sides. A medial constriction occurs on both platform margins. The cusp lies at the posterior platform. It is followed by a short carina extending to the posterior platform end. The free blade occupies 1/2 the whole unit length. It starts from the medial platform, rising evenly anteriorly in height. The keel on the lower surface is slightly bifurcated close to the posterior platform.

**Materials**. 5 specimens from 3 samples.

*Ancyrogondolella* sp. C

Figure 3.11.

**Remarks.** The present specimens differ from most other Early Norian species by the presence of a broad round posterior platform with a length-to-breadth ratio of 1:1. The platform is widest close to the posterior margin, anteriorly it decreases rapidly. There are two to three large denticles on each side of the anterior platform margin. The posterior half of platform margin is flat and unornate. The centrally located small cusp is followed by a large denticle that ends the carina. There is a wide posterior platform brim. On the lower surface, the keel is slightly bifurcated posterior to the centrally located pit.

Materials. 5 specimens from 1 sample.

***Norigondolella*** **Kozur, 1989**

Type species: *Paragondolella navicula steinbergensis* Mosher, 1968.

**Diagnosis.** The genus *Norigondolella* has a platform that extends over the entire unit length or leaves a very short free blade in some specimens. The posterior platform end is round or pointed. Platform margins never develops denticulation, but may be slightly wavy. The evident cusp is located at the posterior platform end or leaves a platform brim. On the lower surface the pit is beneath the posterior platform, surrounded by a wide flaring keel that extends to the whole unit length.

**Comparison.***Norigondolella* is a typical gondolellid platform conodont genus of Norian and Rhaetian time^57^. It differs from the Carnian genus *Paragondolella* in having a flaring basal cavity and an expanded pit on the lower surface of the platform.

*Norigondolella hallstattensis* (Mosher, 1968)

Figures 3.14–3.16.

1968 *Paragondolella navicula hallstattensis* n. subsp.—Mosher^58^, p. 939; pl. 117, figs ?6–9, 10–12.

1980 *Gondolella hallstattensis* (Mosher)—Krystyn^59^, pl. 11, fig. 12.

1991 ‘*Neogondolella’ hallstattensis* (Mosher)*—*Orchard^54^, pl. 4, figs 5, 11.

2018 *Norigondolella hallstattensis* (Mosher, 1968)—Karádi^20^, p. 170, pl. 3, figs 7–8.

2020 *Norigondolella hallstattensis* (Mosher, 1968)—Karádi et al.^24^, fig. 7/20.

**Description.** The P_1_ elements of this species display a broad platform that typically extends to the anterior end of the unit. The posterior platform is rounded and a platform constriction is commonly present on both sides of the cusp. The carina is composed of high and broad denticles that are of nearly same size. The cusp is the last denticle of the carina and is surrounded by the posterior platform. The pit is posteriorly located and surrounded by the keel. Both the keel and the pit are more widely flaring.

**Remarks.***Norigondolella steinbergensis* has a slender platform with sub-parallel margins, and a posteriorly projected cusp located at the posteriormost part of the element.

**Materials**. 16 specimens from 4 samples.

## Methods

Conodont fossils of this study were acquired by the following procedure: the rock samples were dissolved in 10% buffered acetic acid that was changed every 7 to 10 days; residues were sieved into two size classes (N2 mm, N200 μm) and dried at 60 °C; residues were picked systematically from microfossil trays under a stereobinocular microscope and recovered fossils were stored in microcells. Scanning electron microscopy (SEM) was conducted at the Key Laboratory of Stratigraphy and Palaeontology, Institute of Geology, Chinese Academy of Geological Sciences. SEM images were taken at 10 keV on samples coated by gold. All figures and figure legends in this paper are produced by CorelDraw Graphics Suite X7. The URL for this software is www.corel.com/getcoreldraw.
